# Evaluation of convolutional neural network for recognizing uterine contractions with electrohysterogram

**DOI:** 10.1016/j.compbiomed.2019.103394

**Published:** 2019-10

**Authors:** Dongmei Hao, Jin Peng, Ying Wang, Juntao Liu, Xiya Zhou, Dingchang Zheng

**Affiliations:** a College of Life Science and Bioengineering, Beijing University of Technology, Intelligent Physiological Measurement and Clinical Translation, Beijing International Platform for Scientific and Technological Cooperation, Beijing, 100024, China; b Department of Obstetrics, Peking Union Medical College Hospital, Beijing, 100730, China; c Medical Device and Technology Research Group, Faculty of Health, Education, Medicine and Social Care, Anglia Ruskin University, Chelmsford, CM1 1SQ, UK

**Keywords:** Electrohysterogram, Uterine contraction, Convolutional neural network, Maternal health, Monitoring labour

## Abstract

Uterine contraction (UC) activity is commonly used to monitor the approach of labour and delivery. Electrohysterograms (EHGs) have recently been used to monitor UC and distinguish between efficient and inefficient contractions. In this study, we aimed to identify UC in EHG signals using a convolutional neural network (CNN). An open-access database (Icelandic 16-electrode EHG database from 45 pregnant women with 122 recordings, DB1) was used to develop a CNN model, and 14000 segments with a length of 45 s (7000 from UCs and 7000 from non-UCs, which were determined with reference to the simultaneously recorded tocography signals) were manually extracted from the 122 EHG recordings. Five-fold cross-validation was applied to evaluate the ability of the CNN to identify UC based on its sensitivity (SE), specificity (SP), accuracy (ACC), and area under the receiver operating characteristic curve (AUC). The CNN model developed using DB1 was then applied to an independent clinical database (DB2) to further test its generalisation for recognizing UCs. The EHG signals in DB2 were recorded from 20 pregnant women using our multi-channel system, and 308 segments (154 from UCs and 154 from non-UCs) were extracted. The CNN model from five-fold cross-validation achieved average SE, SP, ACC, and AUC of 0.87, 0.98, 0.93, and 0.92 for DB1, and 0.88, 0.97, 0.93, and 0.87 for DB2, respectively. In summary, we demonstrated that CNN could effectively identify UCs using EHG signals and could be used as a tool for monitoring maternal and foetal health.

## Highlights

• A convolutional neuron network (CNN) with an 11-layer Alex-net structure is presented.
• CNN was trained to recognise uterine contractions using an open-access database.
• CNN was also tested on independent EHG data collected by our measurement system.
• CNN model could be used to recognise uterine contractions using EHG signals.

## 1 Introduction

Uterine contractions (UCs) are the result of uterine activity in the form of an action potential and are an important clinical indicator in the processes of labour and delivery [1]. UC monitoring is indispensable in the evaluation of the health of both the mother and foetus by obstetricians [2]. Current methods for measuring UCs include the use of intrauterine pressure catheters (IUPCs) and external tocography (TOCO). IUPCs directly measure the intrauterine pressure changes created by UCs [3], but are limited by their invasiveness and can cause ruptured membranes and infection. TOCO is a non-invasive method of monitoring UC, in which a strain gauge transducer is placed on the mother's abdomen. However, TOCO depends on the subjective criteria of the operator, leading to high between-operator variability [4,5]. Eletrohysterogram (EHG) signals are non-invasive recordings of uterine electrical activity obtained at the abdominal surfaces of pregnant women [5]; they measure the myometrial bioelectrical activity that triggers the mechanical contraction of the uterus [6]. Therefore, EHGs have been used as an alternative for analysing UC.

Various EHG features have been investigated to differentiate UCs and non-UCs between preterm and term labour. It has been reported that several linear features, including the root-mean-square (RMS), median frequency (MDF), and peak frequency (PF), are highly correlated with UCs [[6], [7], [8]]. Combining the dynamic cumulative sum (DCS) with multiscale decomposition is efficient for detecting both frequency and energy changes in EHG signals [9]. Non-linear features, including the correlation dimension (CorrDim), sample entropy (SampEn), Lyapunov exponent (LE), and correlation coefficient H^2^ [10] are also useful for EHG analysis [8,11,12]. The propagation velocity and direction of EHG signals have also recently been proposed as potential discriminators between pregnancy and labour UCs [13,14]. Extracting numerous features from EHG signals provides comprehensive information regarding uterine activity, but it also increases the computational complexity. Therefore, feature selection methods have been applied to eliminate the redundant or irrelevant features when detecting labour [6]. However, previous papers have recognised different features for recognising UCs as being the most important [3,4]. This is partly due to the combination of different feature selection algorithms with different classifiers [6]. Therefore, it is necessary to investigate alternative approaches for recognising UCs, independent of feature extraction and selection algorithms.

Different types of classifiers, including the k-nearest neighbours (k-NN), linear and quadratic discriminant analysis (LDA and QDA), support vector machine (SVM) [15], random forest (RF), and artificial neural network (ANN) [16], have been developed to identify UCs using TOCO, cardiotocograph [17], and EHG signals. However, these classifiers are limited by the K value, sample distribution, or input features. It is often difficult to select the best features or combinational subsets of features for differentiating UCs and non-UCs.

The competitive neural network, recurrent neural network (RNN), and convolutional neural network (CNN) [18,19] have been tested in image recognition and segmentation without additional feature extraction and selection. In particular, CNNs have been used in the recognition of physiological signals, including electromyograms [21,22], electrocardiograms (ECGs), and electroencephalography [23,24]. The advantage of this network is that it can automatically acquire optimal features from training data [25]. Considering the high accuracy of CNNs in detecting ventricular ectopic beats with ECG signals [21,23], their ability to identify UCs using EHG signals should be explored.

The aim of this work was to develop a CNN model for recognising UCs using EHG signals. The performance of the CNN will be evaluated with an open-access EHG database and independent EHG data recorded using our measurement system.

## 2 Materials and methods

**Fig. 1:**
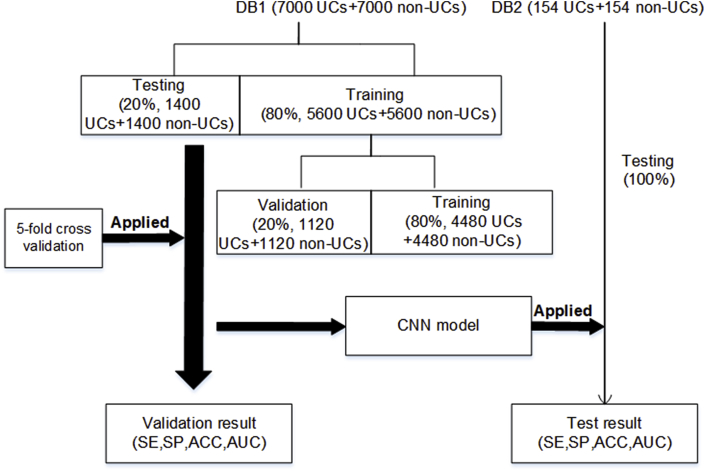
Flow chart of the development of our method *Note: DB1 -* Icelandic 16-electrode EHG database*, DB2 -* our EHG database*, SE - sensitivity, SP - specificity, ACC - accuracy, AUC - area under the receiver operating characteristic (ROC) curve.*

Two EHG databases, i.e., an open-access database (Icelandic 16-electrode EHG database, DB1) [26] and our EHG database (bespoke eight-electrode system, DB2), were used to train, validate, and test the CNN model for identifying UCs. As shown in Fig. 1, five-fold cross-validation was applied to DB1, and then to DB2 to further evaluate the generalisation of the CNN. The details of each step are presented in Fig. 1.

### 2.1 Training and testing using the Icelandic 16-electrode EHG database

#### 2.1.1 Icelandic 16-electrode EHG database

**Fig. 2:**
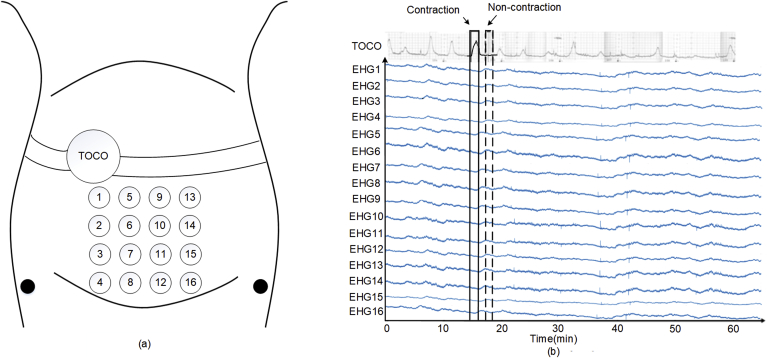
(a) Configuration of the EHG electrodes. (b) Example of 16-channel EHG and TOCO signals from the Icelandic 16-electrode EHG database.

DB1 contained 122 recordings of 16-channel EHG signals from 45 pregnant women, and multiple signals had been recorded for some of these women. The average recording durations for pregnancy and labour were 61 and 36 min. The participants had normal singleton pregnancies without any known preterm birth risk factors. The 16-channel EHG signals were collected from a 4 × 4 electrode grid placed on the woman's abdomen, with the black ground and reference electrodes on each side of the body (not standardised), as shown in Fig. 2(a).

#### 2.1.2 EHG signal pre-processing and segmentation

**Fig. 3:**
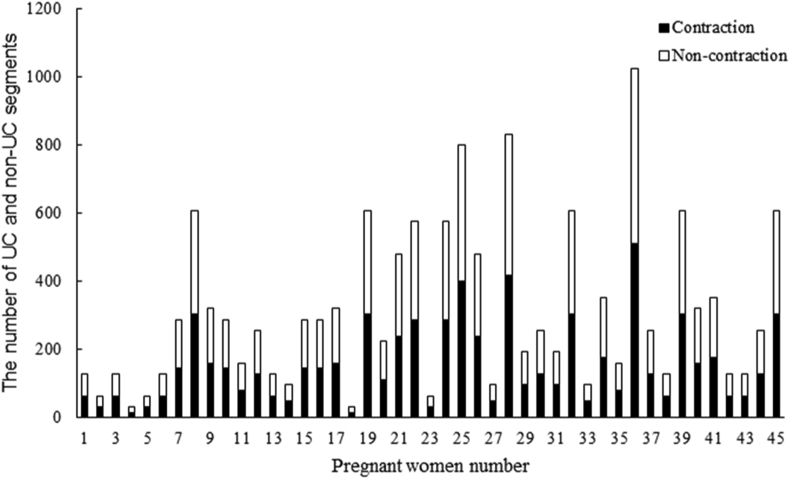
Number of UC and non-UC segments for each of the 45 pregnant women in the Icelandic 16-electrode EHG database.

The EHG signals from DB1 were first pre-processed by a band-pass filter (0.08–4 Hz) [27] to remove the DC components and power line interference. Each EHG signal was then manually separated into UC and non-UC segments based on the UC and TOCO signals. In our previous study, we observed that the UC duration in DB1 was 40–60 s, with an average duration of approximately 45 s. Our clinical experts also confirmed that the UC duration in DB2 was 40–50 s. Therefore, the EHG signal was separated into 45-s segments. If a UC lasted longer than 45 s, only 45 s of the UC was extracted, with its peak corresponding to the TOCO peak. If a UC lasted less than 45 s, it was not used for training and testing CNN. As shown in Fig. 2(b), a 45 s non-UC was extracted between two UCs. In total, 7008 UC and 7008 non-UC EHG segments were extracted from DB1. Fig. 3 shows the number of UC and non-UC segments from each pregnant woman.

Finally, all EHG segments were saved as images, and normalised to 482 × 482 pixels by resizing.

### 2.2 UC classification using the convolutional neural network

**Fig. 4:**
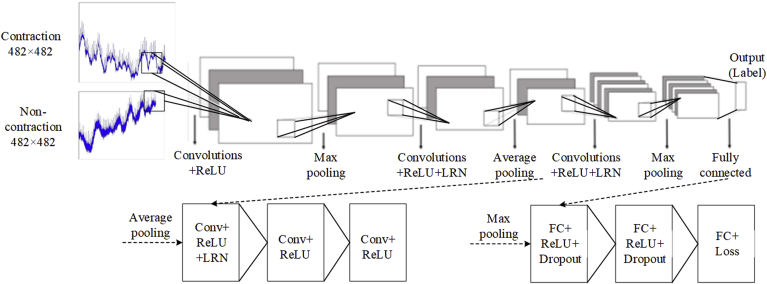
CNN architecture with eleven layers, which are mainly the convolution, max-pooling, and fully connected layers.

As shown in Fig. 4, the CNN used for UC classification consisted of convolutional (Conv), max-pooling, fully-connected (FC), local response normalisation (LRN), dropout, and soft-max layers, and a rectified linear unit (ReLU). As the max-pooling layer is in front of the local response normalisation layer [22,24], using the CNN can reduce the computational complexity and memory size without affecting the recognition capability. The details of the CNN are listed in Table 1.

**Table 1:** Detailed parameters used for all layers in the CNN model.

Layer	Type	Kernel size	Other layer parameters
1	Conv + ReLU	27	Strides = 5, num_output = 96
2	Max-pooling	2	Strides = 3
3	Conv + LRN + ReLU	2	Strides = 1, num_output = 256, local_size = 5, α = 0.0001, β = 0.75
4	Max-pooling	2	Strides = 2
5	Conv + LRN + ReLU	3	Strides = 1, num_output = 384, local_size = 5,α = 0.0001, β = 0.75
6	Conv + ReLU	3	Strides = 1, num_output = 384, pad = 2
7	Conv + ReLU	3	Strides = 1, num_output = 256, pad = 2
8	Max-pooling	3	Strides = 2
9	FC + ReLU +Dropout		Dropout_ratio = 0.5, num_output = 4096
10	FC + ReLU +Dropout		Dropout_ratio = 0.5, num_output = 4096
11	FC + Softmax		num_output = 2, Activation = Softmax

*Pad is the padding number to zero padding.

The main component of the CNN is the Conv layer. The effective features of an input image can be extracted by setting appropriate parameters in the Conv layer, including the image size (l, length; w, width) and the number of filters (m), denoted by l × w@m. Given a convolutional kernel, the output of the Conv layer was calculated according to the following formula [22]:

(1) $$y_{mn}=f\left(\overset{J-1}{\underset{j=0}{\sum }}\overset{I-1}{\underset{i=0}{\sum }}x_{m+i\;n+j}w_{ij}+b\right),\;\left(0\leq m<M,\;0\leq n<N\right)$$

where xis the two-dimensional (2-D) input vector; w is the convolutional kernel with a size of I, J; b is the bias; y represents the output with a size of M,N; andf is the activation function.

The Conv layer extracted features from the input image, the pooling layer down-sampled the feature map and reduced the computational complexity [28,29], and the FC layer exported a 2-D vector to classify UCs and non-UCs.

Every Conv and FC layer was followed by a ReLU function, which was activated by a threshold value without complicated exponential operation [30] and used to quicken the training process [28,29]. The LRN function detected high-frequency features and assigned them with large weights [28].

The filter, weight, and bias of each layer were initialised by searching and manual tuning to achieve an appropriate training time and stability. During the training step, the weight and biases were updated following Eqs (2), (3) [25].

(2) $$w_{l\;}=\left(1-\frac{lr_{\lambda }}{s}\right)w_{l-1}-\frac{lr}{bs}\frac{\partial c}{\partial w}$$

(3) $$\;b_{l\;}=b_{l-1}-\;\frac{lr}{bs}\frac{\partial c}{\partial w}$$

wherew is the weight, b is the bias, l is the layer number, lr is the learning rate, s is the total number of training samples, bs is the batch size, and c is the cost function.

Stride refers to the number of samples that the filter slides over the input signal. A larger stride will result in smaller feature maps, and vice-versa [25]. The parameters in the LRN layer, including the local_size value of 5, α value of 0.0001, and β value of 0.75, were the optimal choices. The kernel size and size of the output neuron (num_output) were set based on generic networks, such as Alex-net [22].

. In the first Conv layer, the size of the input image changed from 482 × 482 to 92 × 92@96 when the kernel was set to 27 and stride was set to 5. The image size then decreased from 92 × 92@96 to 31 × 31@96 after the max-pooling layer. The image was subsequently processed with a stride of 1, kernel of 2, and max-pooling layer to reduce its size from 30 × 30@256 to 15 × 15@256. After the third Conv layer, the image size was further reduced to 13 × 13@384. More features could be obtained from the scanned image when the size of the stride was 1. The Conv and max-pooling layers were designed with output neurons of 13 × 13@256 and 6 × 6@256, respectively. These were followed by FC layers with 4096 neurons. The final FC layer consisted of two neurons, based on Alex-net.

The training algorithm utilised batch normalisation, a loss function, and an activation function, and the stochastic gradient algorithm was used to optimise the weight and achieve better accuracy [31]. The CNN was run using a workstation with a Linux Ubuntu 18.04 LTS Operating System and an NVIDIA 1080Ti GPU. The development environment was the CAFFE-net framework. MATLAB was used for image segmentation, and Python was used for training and testing the CNN.

The main advantages of the CAFFE-net framework and Alex-net structure are as follows [22]:

i. The ReLU has been successfully used as the CNN activation function, and its performance exceeds that of sigmoid function in the deep network;
ii. Dropout is used to randomly ignore some neurons during training to avoid overfitting;
iii. Overlapping max-pooling is used to avoid the blurring effect of average pooling;
iv. LRN is proposed to normalise the local input area and create a competition mechanism for the activities of local neurons, among which the neurons with large responses are activated and those with small feedback are inhibited, thus enhancing the generalisation ability of the model.

#### 2.2.1 Selection of hyper-parameters

**Fig. 5:**
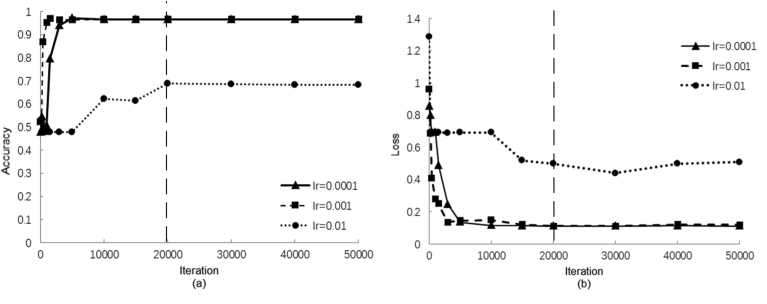
Result of the selection of hyper-parameters for the CNN. (a) Comparison of the accuracies with different learning rates (lr). (b) Comparison of the loss values with different learning rates. Note: *The vertical dashed line represents the stabilisation of both the accuracy and loss values at iteration 20000.*

In this study, the following key parameters were used for the CNN model: mini-batch size = 108, weight decay = 0.0005, learning rate drop factor = 0.1, learning rate drop period = 10, and momentum = 0.9. Their definitions were reported by Kim et al. and Acharya et al. [24,25]. These values were selected as they achieved the lowest loss value and highest accuracy of recognising UCs in a preliminary test that used randomly selected segments (half of the UCs and non-UCs from DB1). The accuracy and loss curves with increasing iterations at different learning rates (lr = 0.01, 0.001, and 0.0001) are shown in Fig. 5. The curves with lr of 0.001 and 0.0001 had lower loss values and higher accuracies than those with a lr of 0.01. Although the accuracies with achieved at lr of 0.0001 and 0.001 were similar, the curve with the lr of 0.001 stabilised earlier than that with the lr of 0.0001. Furthermore, all curves tended to stabilise after 20000 iterations. Based on the results of the preliminary test, fine-tuning was conducted with the lr of 0.001 and maximum number of iterations of 20000 in the subsequent training and testing steps.

#### 2.2.2 CNN evaluation using the Icelandic EHG database

Five-fold cross-validation was utilised to evaluate the performance of the proposed CNN. From the 14016 images in DB1, 14000 were equally divided into five subsets, four of which were used to train the CNN model, and the other was used to test the model. This process was repeated five times. Furthermore, the training set included training (4480 UCs and 4480 non-UCs) and validation (1120 UCs and 1120 non-UCs) data, in which the validation data were used to tune the hyper-parameters of the CNN. The training process ceased at iteration 20000, based on the preliminary test.

ROC and AUC are conventionally used to evaluate the classification performance. Here, the ACC, SE, and SP were calculated as follows [3]:

(4) $$\;Sensitivity=\frac{TP}{TP+FN}$$

(5) $$\;Specificity=\frac{TN}{FP+TN}$$

(6) $$\;Accuracy=\frac{TP+TN}{FP+TN+TP+FN}$$

where TP (true positive) and TN (true negative) are the numbers of UC and non-UC EHG segments that were correctly classified, and FP (false positive) and FN (false negative) are the numbers of UC and non-UC EHG segments that were falsely classified. The results of AUC, ACC, SE, and SP from the five-fold cross-validation were calculated and averaged to evaluate the CNN.

#### 2.2.3 Evaluation of the CNN using the independent EHG database

Twenty women with singleton pregnancies were recruited at the Department of Gynecology and Obstetrics, Peking Union Medical College Hospital, Beijing. All were in term labour with regular UC (37–40 weeks of gestation). The measurements were collected under the Code of Ethics of the World Medical Association (Declaration of Helsinki) and approved by the Research Ethics Committee of Peking Union Medical College Hospital.

**Fig. 6:**
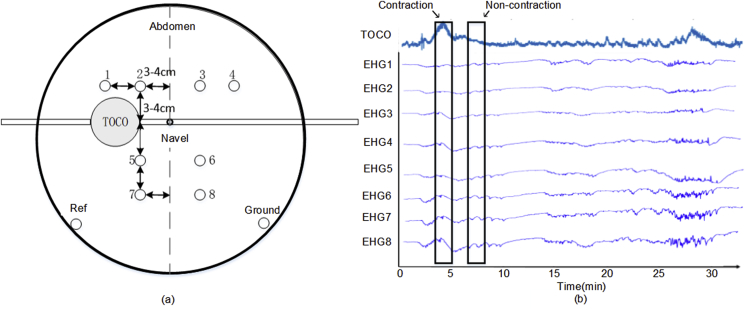
(a) Configuration of the EHG electrodes. (b) Example of eight-channel EHG signals and a TOCO signal from the independent EHG database. *Note: Ref - reference electrode, Ground - ground electrode*.

Eight-channel EHG signals and a TOCO signal were recorded simultaneously using a multi-channel system developed in our lab. The configuration of the eight electrodes is shown in Fig. 6 (a). Electrodes 1 to 4 electrodes were placed on the fundus, electrodes 5 and 6 symmetrically placed below the navel, electrodes 7 and 8 were placed on the uterine cervix, and the reference and ground electrodes were placed on each side of the iliac crests. Disposable EHG electrodes (L-00-S AMBU Denmark) with a size of 68.2 × 55 mm were used. A TOCO transducer was also attached to the surface of the pregnant woman's abdomen. The EHG and TOCO signals were sampled at 250 Hz for 35 minas. The pregnant women marked UCs when they felt contractions during recording.

The EHG signals of DB2 were pre-processed and segmented in a similar manner to those of DB1. Fig. 6(b) provides one example of UC and non-UC segments in DB2. In total, 154 UC and 154 non-UC EHG segments were obtained to further test the CNN developed using DB1.

## 3 Results

### 3.1 Evaluation of the CNN model using DB1

**Fig. 7:**
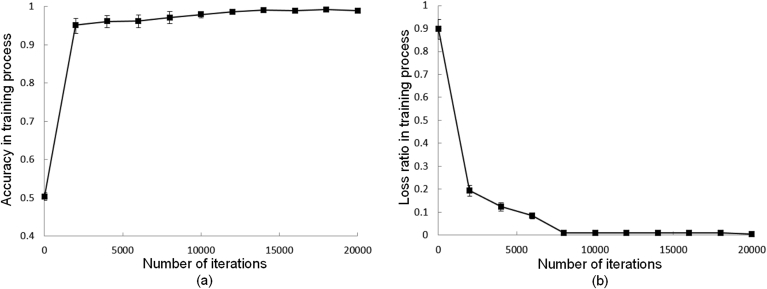
(a) Changes in accuracy with the number of iterations. (b) Changes in the loss ratio with the number of iterations (step of 2000). The mean and standard deviations from the five-fold cross-validation are also provided.

Fig. 7 shows the accuracy and loss ratios that were evaluated using the validation data (1120 UCs and 1120 non-UCs). At iteration 20000, the accuracies of the five validations were 98.54%, 99.14%, 99.40%, 98.75%, and 98.56%, and the loss ratios were 0.11%, 0.89%, 0.12%, 0.09%, and 0.11% respectively.

**Fig. 8:**
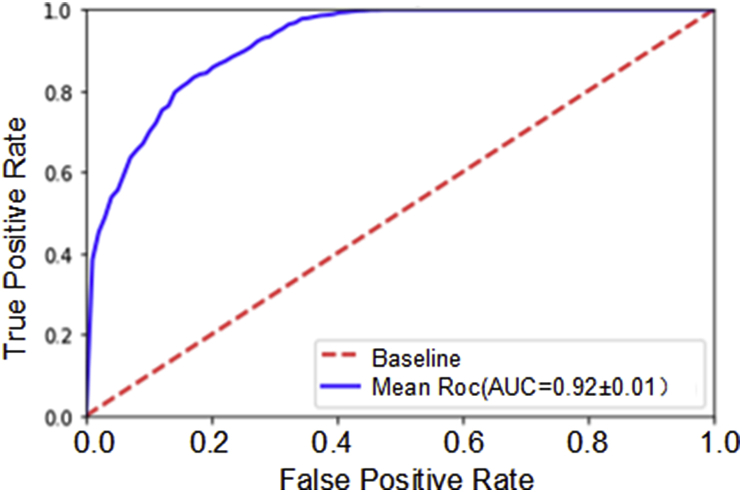
ROC curve for recognising UCs in DB1 from five-fold cross-validation.

Table 2 provides the separate test results from folds 1–5 of DB1. The result of each fold was similar, indicating the reliability of the CNN model. Overall, the CNN model achieved an SE of 0.87, SP of 0.98, ACC of 0.93, and AUC of 0.92 from DB1 (Table 2 & Fig. 8).

**Table 2:** Testing result from folds one to five of DB1.

Database		Resulting values				Calculated parameters		
		FP	FN	TP	TN	SE	SP	ACC
DB1	Fold1	20	169	1222	1360	0.88	0.99	0.93
	Fold2	23	176	1205	1357	0.87	0.98	0.93
	Fold3	26	180	1211	1354	0.87	0.98	0.93
	Fold4	30	190	1201	1350	0.86	0.98	0.92
	Fold5	22	188	1203	1358	0.86	0.98	0.92
	Overall	121	903	6042	6779	0.87	0.98	0.93

### 3.2 Evaluation of CNN model using DB2

As the results of the five folds were similar, the CNN model developed from fold1 was tested using DB2, which achieved an SE of 0.88, SP of 0.97, ACC of 0.93, and AUC of 0.87, with corresponding FP, FN, TP, and TN of 5, 18,136, and 149, respectively.

## 4 Discussion

Monitoring UC with EHG signals is non-invasive, low-cost, and simple, and provides a great opportunity for developing long-term, wearable UC-monitoring systems both at clinics and at home. In this study, we developed and evaluated a CNN model for identifying UCs from EHG signals. To the best of our knowledge, the CNN was first developed for classifying EHGs, and the parameters of all layers of the CNN model were specifically investigated to identify UCs using EHGs.

CNNs were originally designed for evaluating images. Therefore, many of their properties are specific for image representations, and there is a mature platform for tuning CNN parameters for 2-D image classification. Though one-dimensional (1-D) CNNs have been recently applied for classifying time-series (such as ECG signals) [23], the 2-D CNN method is more suitable for small datasets than 1-D CNNs [32,33]. Therefore, in our first test, the EHG signal time series was segmented and converted into an image to input into the CNN. We validated and initially tested the CNN model using DB1, and achieved an SE of 0.87, SP of 0.98, ACC of 0.93, and AUC of 0.92, and the corresponding values were 0.88, 0.97, 0.93 0.87 for DB2, respectively. As shown in Table 3, Khalil [9] achieved an ACC of 0.93 when monitoring UCs, and Muszynski [10] obtained an ACC of 0.96 when employed the nonlinear features of EHG signals. However, the main goal of their studies [9,10] was to differentiate UCs between different types of pregnancies or at-risk pregnancy, while our study aimed to differentiate between UCs and non-UCs. Our proposed method may be improved by introducing DCS, H2 correlation coefficient, fusion, and elimination steps [10]. The CNN does not require additional steps for EHG signal feature extraction and selection, thus, signal processing is simpler than that of conventional machine learning algorithms. This is because the feature extraction, selection, and classification procedures are merged into a single CNN structure [34]. From the results of our study, it can be concluded that CNNs can be used to identify UCs using EHG signals.

**Table 3:** Summary of the classification results achieved by different studies.

Ref.	Data source	Feature/method	Classifier	Result (Highest value)
				SE SP ACC AUC
[9]	Independent database with 32 recordings		DCS algorithm	0.93
[10]	Independent database with 51 recordings	Nonlinear correlation coefficient H^2^		0.96
Current study	Icelandic 16-electrode EHG database		CNN	0.87 0.98 0.93 0.92
	Independent database from 20 pregnant women		CNN	0.88 0.97 0.93 0.87

Cross-validation has been widely used to test and evaluate the generalisation of CNN models [25,28,35]. In previous work, the generalisation of classifiers was evaluated using the same data source. However, in this study, the CNN model was evaluated using both an independent database (DB2) and the original database (DB1). DB2 was collected from different races in a different hospital with a different recording protocol to DB1. Merging two databases could increase the sample size, which would be better for training the CNN model. However, DB1 is an open-access database with 14000 segments from 122 recordings, while DB2 only provided 308 EHG segments from 20 recordings. The impact of the small size of DB2 can be neglected due to the large difference in the sample sizes of DB1 and DB2. However, if the data from DB2 are treated as an independent test set, they can be used to evaluate the generalisation of the CNN model. Additionally, EHG signals from different recording systems may generate different signal features, depending on the technical specifications of the system. Moreover, the electrode position of our EHG system was different to that of the Icelandic 16-electrode system. Finally, the results from DB2 could be used to guide our future research for developing a specific CNN model for our system.

With the increasing demand for healthcare during pregnancy [36], CNN models for identifying UCs have prospective clinical applications. If the CNN model is further improved, the EHG segments corresponding to UCs could be recognised more accurately. Therefore, in clinical practice, an automatic identification system could be utilised for long-term wearable UC monitoring. Further, the UCs leading to labour could be distinguished from non-labour UCs, which will improve the diagnosis of preterm labour. Our clinical experts agree that our proposed method is very promising for clinical practice.

In future studies, the effect of UC duration on the CNN classification results will be investigated [34,35], and the performance of 1-D and 2-D CNNs will be compared for this specific clinical application. More EHG signals will be recorded to improve the recognition ability of the CNNs.

## 5 Conclusions

This study demonstrated that the CNN could be used to recognise UCs efficiently from EHG signals. With this method, UCs can be detected reliably and accurately, providing a novel approach for monitoring labour progress, and maternal and foetal health.

## Data availability

The database used in this study can be accessed via: https://physionet.org/pn6/ehgdb/.

## Ethical approval

The analysis of this database was approved by the Research Ethics Committee of the Faculty Research Ethics Panel (FREP) of Peking Union Medical College Hospital and Anglia Ruskin University.

## Conflicts of interest

All authors declare that they have no conflict of interest.
